# Effective index model as a reliable tool for the design of nanostructured thin-film solar cells

**DOI:** 10.1038/s41598-023-33085-3

**Published:** 2023-04-17

**Authors:** P. A. Sánchez, O. Esteban, M. H. Elshorbagy, A. Cuadrado, J. Alda

**Affiliations:** 1 Photonics Engineering Group, University of Alcalá de Henares, 28801 Alcalá de Henares, Madrid Spain; 2 Physics Department, Faculty of Science, Minia University, El-Minya, 61519 Egypt; 3 Escuela de Ciencias Experimentales y Tecnología, University Rey Juan Carlos, 28933 Móstoles, Madrid Spain; 4 Applied Optics Complutense Group, Faculty of Optics and Optometry, University Complutense of Madrid, C/Arcos de Jalón, 118, 28037 Madrid, Spain

**Keywords:** Energy science and technology, Mathematics and computing, Nanoscience and technology, Optics and photonics, Physics

## Abstract

Nanostructured anti-reflection coatings (ARC) are used to reduce the reflectivity of the front surface of solar cells. Computational electromagnetism helps to evaluate the spectral reflectivity of of this type of ARC using several approaches. They typically require large computational resources both in time and hardware elements (memory allocation, speed of processors, etc.). Long computational times may jeopardize optimization processes based on the iterative evaluation of a given merit function that depends on several parameters. Then, simplified analytic methods can speed up this evaluation with moderate computational resources. In this contribution we adapt an Effective Index Model (EIM) to the case of the design of an ARC made with nanoparticles (NP) embedded in a medium at the front surface of a thin-film silicon solar cell. Our approach modifies the discrete dipole approximation method to adapt it to the geometric and material properties of the NPs. The results obtained from the analytic method are compared with those evaluated through a Finite Element Method (FEM) for several cases involving variations in the size and geometry of the NP arrangement, obtaining reflectances that differ less than 10$\%$ for the worst case analyzed but bieng about 100 times faster than the FEM.

## Introduction

The use of fossil energy reservoirs has also produced a climate change crisis triggering both natural, social, and economic disturbances^1^. All these issues have caused a growing interest in the research and development of sustainable forms of energy^2–5^. There exist many types of renewable energy: solar, wind, biomass, etc. Among these options, solar energy is one of the most widely used. It is based on a reliable technology and presents several advantages when compared to other renewable sources^6^. Since the appearance of the first silicon solar cell in 1954^7^, we have witnessed successive improvements in efficiency, that reaches values over 40% for multi-junctions cells^8^, which represent the trend of the technology. However, a more humble solution, based on thin-film silicon solar cell, is very well positioned in terms of cost/watt ratio^9,10^ and easiness of the fabrication process^11^. The low efficiency of thin-film silicon solar cells is mainly caused by the thin active layer, which diminishes the probability of absorption of the incoming photons^10–13^ and the high reflectivity of this arrangement—as a result of the combined effect of the interfaces and being the back contact metal layer quite close to the cell surface. However, the thin-film configuration reduces the electron–hole recombination rate, increasing the probability of the charge-carries to reach the extracting contacts^14^.

To improve the efficiency of solar cells, several strategies applicable to thin-film designs have been proposed. For example, the top or bottom contacts of the cell can be nano-textured which either random or periodic distributions^15–17^. Also, the top surface can incorporate multilayer planar antireflection coatings (ARC)^18^, etc. Recently some advances have been reported^17,19–22^ for the use of nanoparticles (NPs) as elements included into the ARC, as well as integrated into the back contact, and even inside the active layer. When these NPs are metallic, the solar irradiance excites Localized Surface Plasmon Resonances (LSPR). This makes possible the manufacture of filters and, with an adequate positioning and dimensioning of the NPs in the solar structure, devices that could be included within the so-called plasmonic photovoltaics^2,23,24^. The design of optical metasurfaces working as antireflection coatings in energy harvesting applications is a fundamental task for improving the overall behavior of solar cells, especially when texturization is not a suitable option.

Planar ARCs based on embedded nanoparticles have some advantages compared to other approaches. When properly designed, they trap the light within a broadband range, and redirect it towards the active layer of the cell improving its harvesting efficiency^11,24–26^. An optimum design of these nanostructured ARCs is not easy, and requires dedicated modeling and simulation before fabrication. A typical approach to the computation evaluation of the performance of these structure is made through computational electromagnetism packages that evaluate the propagation of light and the interaction with material structures. This approach makes a complete characterization of the electromagnetic wave, and it is also named as a Full-Wave (FW) calculation. These algorithms evaluate Maxwell’s equations within a volume properly meshed to comply with the conditions of the method. Due to the fine meshing associated with the subwavelength geometries of NPs, the simulation becomes very demanding in computational resources, and takes a quite long time to produce valid results. Besides, to properly reproduce the structures under analysis, most of the times, the calculation has to be done within in 3D. Some alternative analytic models have been already proposed to ease the simulation effort. In this paper we will use the concept of Effective Index of refraction Model (EIM), where the whole metasurface is replaced by an homogeneous layer with equivalent optical properties, paying special attention to the replication of the Fresnel coefficients of the structure. This approach renders models suitable for ordered arrays^27–29^ or random distribution of NPs inside a host matrix^24,29–32^, within a range in NP’s size, and volume fraction. These previous analysis give coincident qualitative results for the spectral reflectivity, that is the parameter of interest for ARC located on top of a solar cell made of a high-index semiconductor. The calculated reflectivities depart from those calculated with FW tools when the NP becomes larger than the Rayleigh limit ($\sim 40$ nm) or the packaging of them is dense^31,33^. Also metallic NP behave differently under the EIM showing variations that depend on the material and geometry of the NP and the given arrangement. In such cases, the multipolar terms of the NP mutual interaction, substrate effects, and dynamic depolarization become significant and the dipole approximation begins to fail. This behavior limits the applicability of the analytic method to low coverage ratios^31,34^, or small NPs^34^

In this work, we compare the reflectance numerically calculated through FW calculations with the value obtained using the EIM that is based on the Discrete Dipole Approximation (DDA). This analysis helps to understand the limitations when replacing the FW approach by the DDA method. These results can be considered as a promising result for the development of a simple design methodology, which will require validation from experimental measurement in the future. In fact, the DDA analytic model simplifies the design process. Therefore, it reduces computation time in several orders of magnitudes, minimizes the required memory allocation, and doesn’t need high-end processors. The model can be coded in Matlab or Python, allowing its modification and extension to more complex structures, and its implementation within complex optimization algorithms. This is of great importance when the geometry and material choices of the metasurface are implemented in an optimization process where a predefined merit function needs to be evaluated hundreds, or thousands, of times before reaching the desired performance^35–37^. The manuscript is organized as follows: “Reflectance calculation for 2D metasurfaces” defines the studied structure, with special focus on the geometric arrangement of the proposed ARC structure. Section “Reflectance calculation obtained through FW and effective index model approaches” analyzes the differences between the obtained reflectance using the analytic model and the output of the FW one. In this case, special care has been paid to highlight the spectral difference, both in shape and magnitude. Finally, Section “Conclusions” presents the conclusions of this work

## Reflectance calculation for 2D metasurfaces

A careful designed ARC improves the overall efficiency of a thin-film based solar cell. The performance of the ARC is mainly given by the value of its reflectance, that should be minimized as much as possible, maintaining the absorption low at the ARC layer, and tuning the ARC spectral transmittance to the absorption bandgap of the active layer. The optimization of the optical behavior should also maintain a good electric conductivity of the top contact of the solar cell. Therefore, reflectivity becomes the figure of merit of the optimization process.

**Figure 1:**
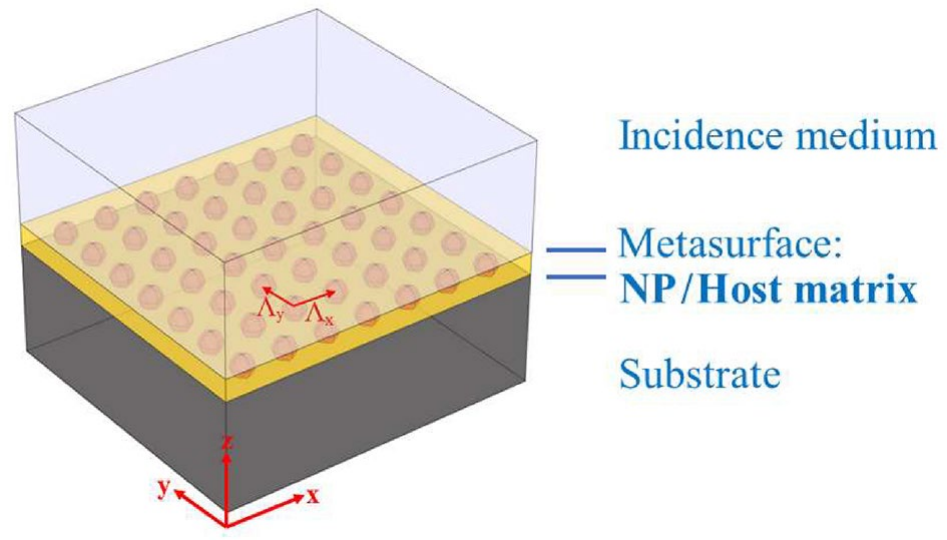
Basic multilayer structure with a metasurface used for the test.

**Figure 2:**
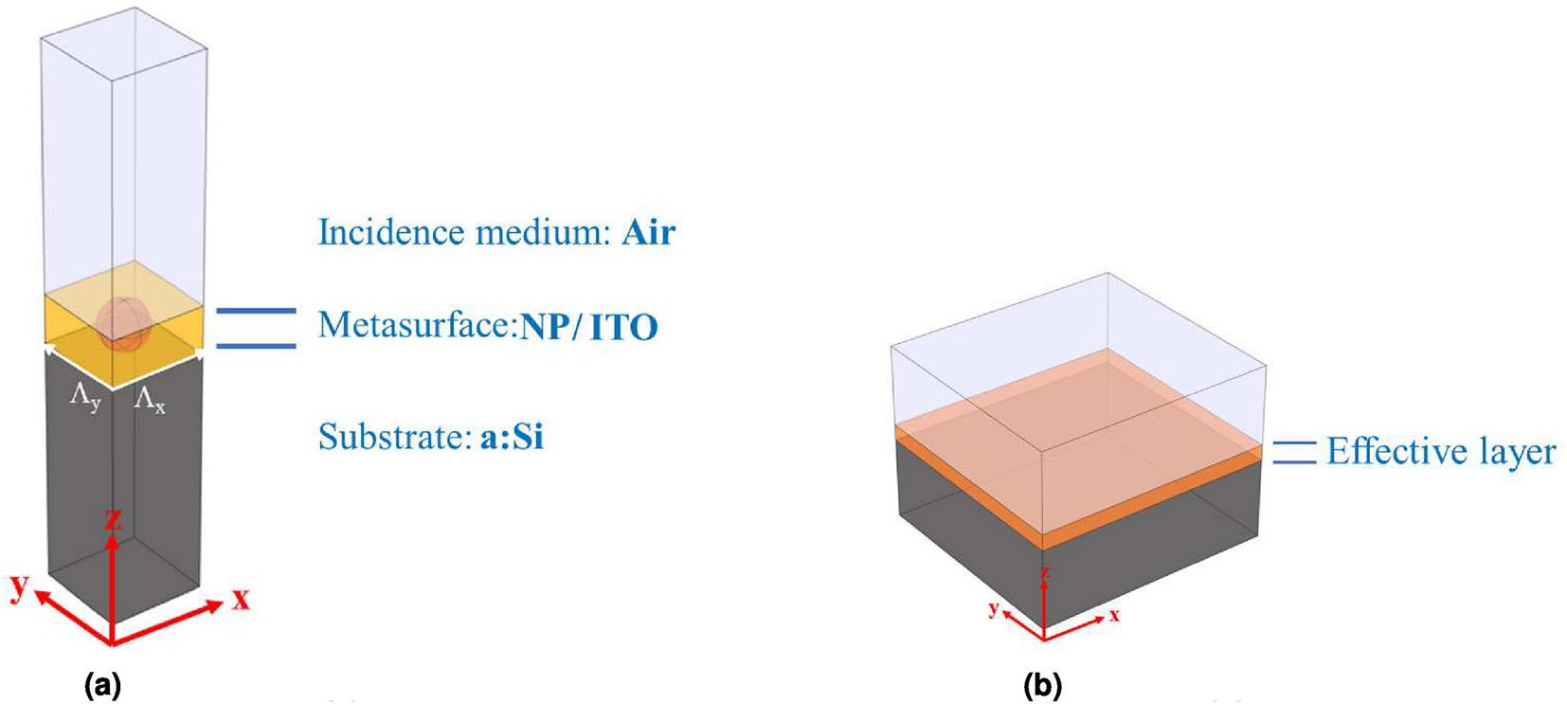
(**a**) Unit cell used for FW calculation based on numerical FEM methods. (**b**) Equivalent multilayer with effective metasurface layer.

Figure 1 represents a basic layout of the ARC that we consider in this contribution. It contains a 2D nanostructure metasurface sandwiched between two homogeneous semi-infinite media that contains a regular arrangement of NPs. It is important to note that the model presented in Fig. 1 is a simplification of a thin-film solar cell. A more realistic model would include a much more complex structure, even with auxiliary layers between the top layer and the active layer (n-a:Si or p-a:Si). These additional layers can be considered by including their characteristic matrices into the EIM. In this work, the spectral reflectance—a key performance indicator of the solar cell—has been evaluated when considering the addition of NPs to the front layer. The optical properties of the metasurface depends on the geometry of the NPs (individual size and type of arrangement), and the optical properties of the material of the NP and the hosting medium. In our case, the hosting medium is the transparent oxide acting as the top contact of the thin-film solar cell. The calculation of the reflectance of this ARC can be done by several ways. One of the most widely used method is based on a FW simulation that uses a Finite Element Method (FEM). This method solves Maxwell’s equations on a mesh adapted to the geometry and dimension of the domain of interest. The typical way of simulating the metasurface requires the definition of a unit cell that is replicated laterally by using periodic boundary conditions. In this work we have used Comsol-Multiphysics to render quite accurate solutions. Thus, these results are obtained at the expense of high computational costs, in the form of high performance computers, long calculation times, or both. However, these requirements can be relaxed by considering an analytic approach where the nanostructure metasurface is replaced an equivalent layer having the same optical properties. Therefore, the complex—although accurate—calculation made using FEM is simplified as a multilayer approach (see Fig. 2a). The key point of this replacement is the appropriate definition of the geometrical and optical properties—the effective index of refraction, $n_{\text{eff}}$, and thickness—of the equivalent layer.

The simplest way to obtain an effective medium replacing the mixture of nanoparticles inside a host medium, is based on the Maxwell–Garnett approach^34^. However, the suitability of this model is limited to random distribution of NPs in a dilute concentration so that the mutual interactions are negligible. When the interaction between NPs and the effect of the substrate are relevant, some other approaches should be considered, such as FW or Discrete Dipole Approximation (DDA) In this case, an EIM can be obtained through the DDA (see Fig. 2b). This model considers each NP as an electric dipole, and the overall performance of the equivalent layer includes the interactions among the dipoles in the array. However, this equivalent layer is no longer isotropic due to the substrate proximity effect. In fact, each NP has an associated dipole plus its own image within the substrate, which means that the effective dielectric tensor is diagonal and contains two parallel components and one perpendicular with respect to the interface^29^. An additional correction must be also made when the NP size is beyond the Rayleigh limit (radius larger than 40 nm)^31,38^. In this case, the quasi-static approximation is no longer valid^39,40^, and the NP polarizability should include dynamic depolarization and radiative damping effects. This correction is known as the Modified Long-Wavelength Approximation (MLWA)^41^, and it will also be used in this work to modify the DDA model with which it will be built in EIM.

Although not directly based on the effective index approach, the Coherent Scattering Model (CSM) allows to obtain the surface reflectance from arrayed structures^42^. It employs the rigorous multiple-scattering theory to derive reflection and transmission coefficients at each interface to be utterly employed in a Fresnel formalism. By using the diagonal elements of the scattering matrix, it evaluates the average of the electric field as a superposition of the incident and the radiated by the nanoparticles, considering them as point scatterers.

When comparing the DDA and the CSM models, in the limit of dilute concentration up to a surface coverage of about 5$\%$, and with NPs radius up to 60 nm, the calculated reflectances coincide for normal incidence conditions, which is the case for solar cells, as described by the international and European standards ISO 9050 and EN 410. Therefore, we restrict ourselves to normal incidence conditions and we use the modified DDA approach to obtain a spectral reflectance to be compared with the results from a FW calculation. A first advantage of the equivalent layer model is that it reduces the dimensionality of the problem, moving from the 3D geometry of the FW calculation domain to the 2D case for the multilayer structure, which implies a global reduction of computation times, not only due to the reduction of dimensions, but also because its implementation is simple, reducing computational costs as well. A detailed derivation of the effective index model under the DDA approach can be found in the Supplementary data. But in short, from Supplementary Eqs. (S1)–(S4), it is possible to obtain the effective dielectric permeabilities and the effective parallel and perpendicular poralizability. Adding the MLWA correction from Supplementary Eqs. (S9)–(S10), it is feasible to get the effective dielectric permittivity of the metamaterial layer^43,44^, and then its complex refractive index components as $n_{\text{eff}}=n-ik=\sqrt{\varepsilon_{\text{eff}}}$, either parallel or perpendicular to the interface with the substrate. Once obtained this complex effective refractive index, it can be used to evaluate the reflectance of the structure through a T-matrix method^29,45^, where the metasurface is replaced by a homogeneous layer.

## Reflectance calculation obtained through FW and effective index model approaches

When calculating the reflectance of a metasurface composed of NPs embedded into a host matrix on top of a suitable substrate, the main parameters affecting the spectral reflectance are the geometrical arrangement of the NPs inside the composite layer, the refractive indices on the NPs and host matrix respectively, and the shape and size of the NPs themselves. For the sake of simplicity, the NPs will be considered as spheres and arranged in a square grid. Furthermore, the host matrix medium—Indium Tin Oxide (ITO), the external one—air, and the substrate—amorphous silicon, a:Si—are fixed in the structure. In our analysis we have also calculated the optical response for two dielectric materials: SiO$_2$ and AlN; and three metallic nanoparticles: Ag, Au and Al. We have found that the results are alike. Therefore, we have illustrated the application of the EIM to the case of SiO$_2$ for the dielectric case, and Ag for the metallic one (Although some examples of results obtained for other materials can be found in the Supplementary data, in Figs. S1 and S2). All the refractive indices have been extracted from^46^ and have been used in both FW and EIM reflectance calculation. The remaining geometric parameters to minimize the reflectance are: the NP radius that varies in the range $\rho _{\text{NP}} \in [30, 60]$ nm, and the period of the square grid, $\Lambda$, that is given as a multiple of $\rho _{\text{NP}}$, as

1 $$\begin{aligned} \Lambda = a \rho _{\text{NP}}, \end{aligned}$$

with $a \in [4, 8]$. The relation between the period $\Lambda$ and the radius of the $\rho _{\text{NP}}$ allows the definition of the area coverage ratio as:

2 $$\begin{aligned} C_a = \frac{\pi \rho ^2_{\text{NP}}}{\Lambda ^2}=\frac{\pi }{a^2}. \end{aligned}$$

After combining Eqs. (1) and (2), we find an area coverage depending on the value of *a* in Eq. (1) that reaches up to 20% for $a=4$, which is larger than values previously reported^29,31,34,47^.

### Dielectric NPs embedded into an ITO matrix

**Figure 3:**
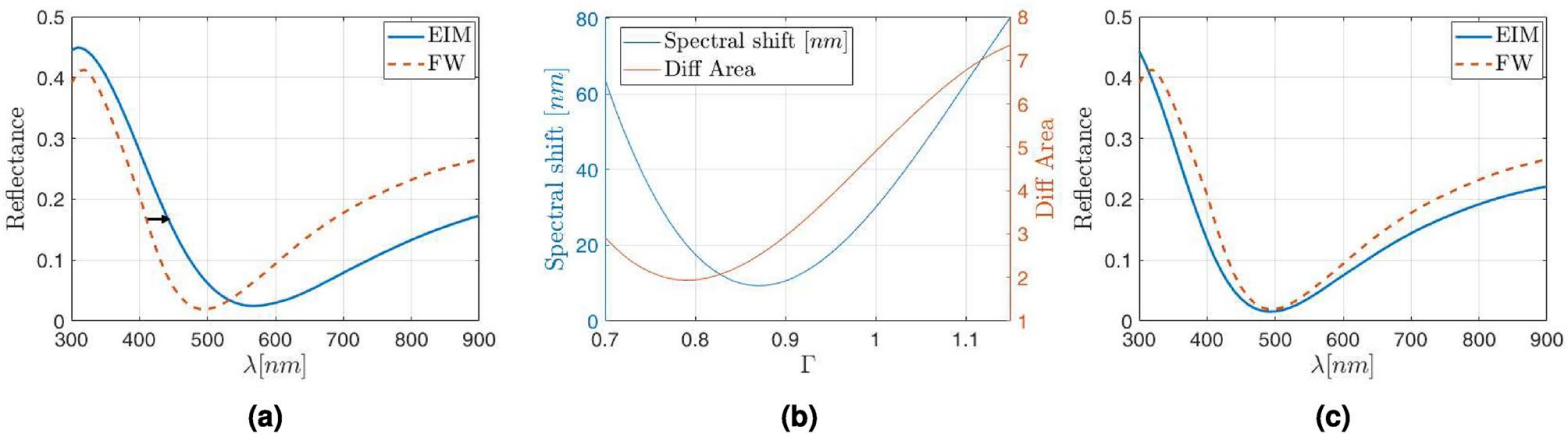
(**a**) Spectral shift (marked with an arrow towards the right) between the FW (red dashed line) and EIM model (blue solid line) for SiO$_2$ NPs embedded into ITO with $\rho _{\text{NP}}$ = 30nm and $\Lambda =8\rho _{\text{NP}}$. (**b**) Evolution of the redshift (red solid line) and difference of areas under the reflectance curves (blue solid line) as function of the correction factor applied to the effective thickness. (**c**) Reflectance obtained with the FW (red dashed line) and EIM model (blue solid line) after applying the constant correction factor $\Gamma =0.83$.

Our first case considers SiO$_2$ NPs having a radius $\rho _{\text{NP}}=30$ nm, and arranged as a square lattice with period $\Lambda =240$ nm (meaning $a=8$), embedded in an ITO matrix. This arrangement renders a surface coverage of about 5% which is similar to some other values presented in literature^31,47^. The reflectance of this structure obtained through the FW method and the EIM, are plotted in Fig. 3a, where the FW methods corresponds with the dashed red line, while the EIM appears as a solid blue line. As can be seen, the spectral shape is quite similar when comparing both methods. However, a slight spectral shift appears, with a redshift of the EIM result respect to the FW one, which is considered closer to the actual one.

The calculation of the reflectance for an equivalent index multilayer makes uses of the Fresnel formalism. Then, the key parameters are the refractive index and thickness of each layer. The value of the thickness of the equivalent layer has been considered equal to the physical thickness, $d_{\text{phy}}$ that we consider equal to the diameter of the NP, $d_{\text{phys}}= 2\rho _{NP}$. Although the value of the thickness of the layer appears in the analytic formalism of the DDA, it has very little influence in the final value obtained from the EIM. However, the thickness of the layer affects to the Fresnel reflectances in a multilayer calculation. Therefore, we have defined an effective thickness as

3 $$\begin{aligned} d_{\text{eff}}=\Gamma d_{\text{phy}}. \end{aligned}$$

Using the parameter $\Gamma$ defined in Eq. (3) we can evaluate the spectral shift of the reflectance *vs.* $\Gamma$. This dependence opens the way to use the effective thickness $d_{\text{eff}}$ as an additional parameter to better fit the reflectance, where the layer equivalent to the metasurface is characterized by $n_{\text{eff}}$ and $d_{\text{eff}}$. The effective thickness can be used to reduce the spectral shift, or to minimize the area between the FW reflectance and the curve obtained through the EIM formalism. By computing these differences between both models, namely redshift and area between the reflectance curves, we can obtain the curves plotted in Fig. 3b, where the left scale shows the redshift of the reflectance curves (blue solid line) while the right scale shows the differences in the areas under such reflectance curves (red solid line). From Fig. 3b, two different values for $\Gamma$ minimize the differences between reflectances, depending on the adopted criterion: $\Gamma =0.79$ when reducing the spectral shift, and $\Gamma =0.87$ when minimizing the difference in area between the reflectance curves. Since both criteria show different values, for the sake of simplicity, we have considered the average between both values. Therefore a value of $\Gamma =0.83$ has been used to correct the effective thickness of the equivalent layer. The result is plotted in Fig. 3c. It is straightforward to see that in this case, the reflectance minimum coincides for both methods around 490 nm, and the global difference between both methods—defined as the absolute diference between both curves—is on average below 5%, that is half of the error foreseen when using a DDA approach to get the effective index of a metasurface.

**Figure 4:**
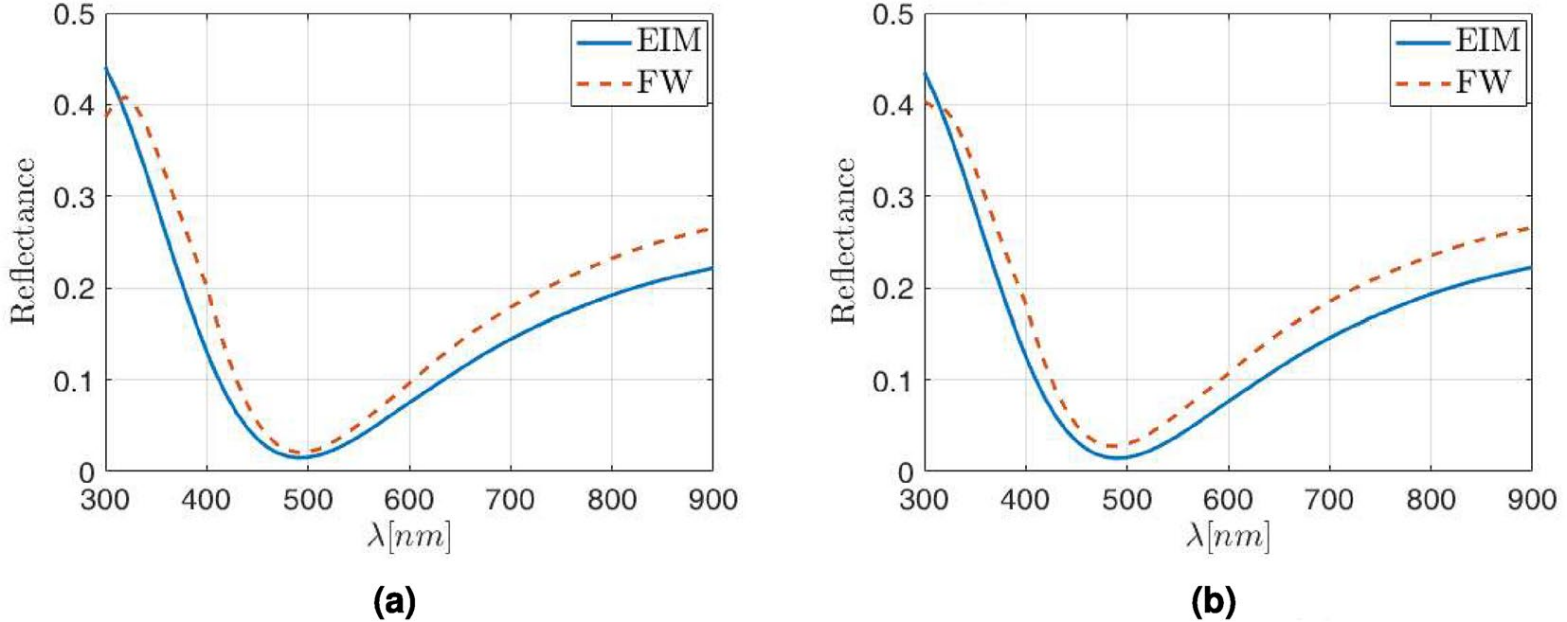
Reflectance obtained with the FW (red dashed line) and EIM (blue solid line) model when (**a**) the lattice parameter is $\Lambda =6\rho _{\text{NP}}$. (**b**) The lattice parameter is $\Lambda =4\rho _{\text{NP}}$. Both cases with the correction factor $\Gamma =0.83$ applied to the physical thickness of the metasurface layer.

The same procedure has been used to obtain the reflectance for higher density packaging (lower value of *a* in Eq. (1)). Indeed, if the lattice parameter reduces either to $\Lambda =6\rho _{\text{NP}}$ (which means a surface coverage of about 9$\%$), or even to $\Lambda =4\rho _{\text{NP}}$ (in this case the surface coverage increases up to about 20$\%$, much larger than those reported in the literature for square arrangements), while keeping the NP radius at 30 nm, the correction factor is constant: $\Gamma =0.83$. The reflectances calculated for these cases are shown in Fig. 4a,b.

**Figure 5:**
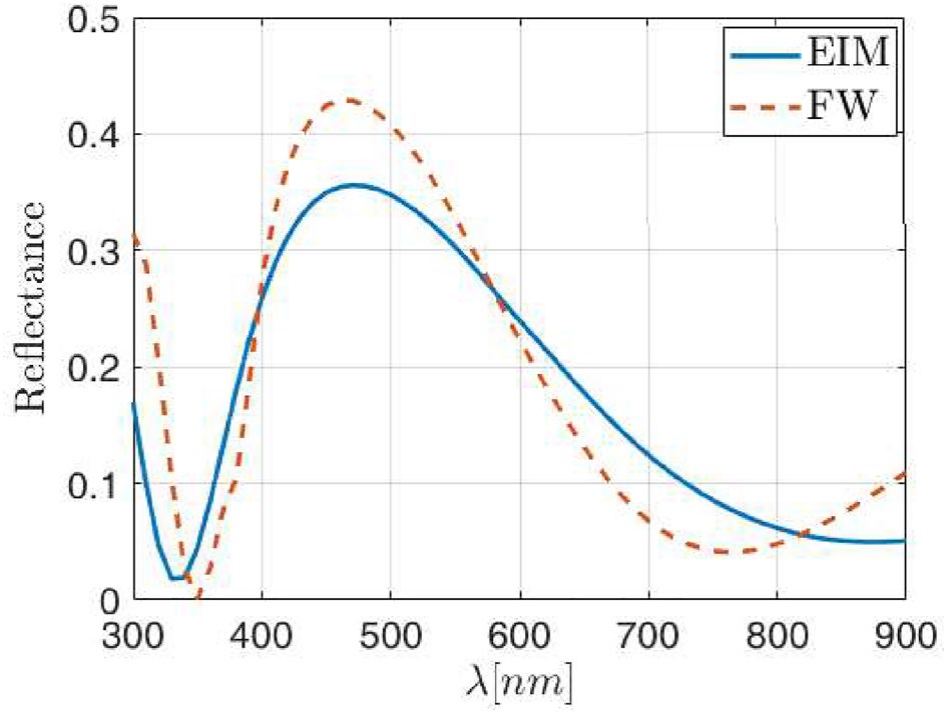
Reflectance obtained with FW (red dashed line) and EIM (blue solid line) when the SiO$_2$ NP radius increases up to 60 nm, with a lattice parameter of $\Lambda =4\rho _{NP}$ and a thickness correction factor $\Gamma =0.83$.

Again, the corrected EIM reflectance agrees with the FW one with an accuracy better that 5%, being the larger discrepancies at the long wavelengths tail, beyond 750–800 nm. This good fitting happens even beyond the limit of dilute metamaterial (with a surface coverage of about 9$\%$). An additional check can be done if the NP radius increases up to $\rho _{\text{NP}}=60$ nm, which is in the limit of that could be considered as a nanoparticle^48,49^. This value leads to a relatively thick metasurface with a physical thickness of $d_{\text{phys}}=120$ nm. When using these values to calculate the reflectance, interferometric-like patterns are obtained. This behavior is observed in Fig. 5, where the reflectance is calculated with both the FW (red solid line) and the EIM (blue dashed line) with the same effective layer thickness correction of $\Gamma =0.83$ and a lattice parameter of $a=4$ (the higher density package studied here). As seen so far, there is a good agreement between both methods, with similar reflectances for all cases. In the case the high density packaging ($a=4$), the error slightly increases at specific wavelengths, although it remains below 7%. This behavior is kept when the lattice parameter increases (for $a=4$ and $a=6$).

Therefore, the thickness correction factor can be considered as a way to easily correct the systematic drift in reflectance when using dielectric NPs inside a host matrix. In this case the EIM can be advantageously used in the optimization of antireflective coatings based on metasurfaces. Our results show a good agreement between the equivalent index model with the FW model. EIM renders accurate enough values while reducing computational execution times up to 2 orders of magnitude for all the cases studied in this contribution (see Supplementary Tables S1 and S2), It also can include optical constants as input values for other numerical solvers describing the electric behavior in solar cells. This improvement comes from the differences in the performance of the numerical and analytical models. While the former use partial differential equations to solve a complex model, with the large consumption of resources that this implies, the analytical models are based on solving much simpler equations on a quite limited number of interfaces. The cost reduction comes specifically from the capacity of the presented model to transform a complex problem into simple matrix operations, that can be easily implemented in a matrix calculation environment as MATLAB, which is specialized in these tasks.

### Metallic NPs embedded into an ITO matrix

After applying the same methodology described in “Dielectric NPs embedded into an ITO matrix”, a quite different results is obtained when silver NPs are used. The difference is mainly caused by the generation of localized surface plasmon resonances in the visible range under normal incidence. The excitation of these plasmons increases the overall performance of the cell if they are very close to the active layer, less than 10 nm^50,51^. The structure is composed of Ag NPs 30-nm in radius and having a square lattice parameter $\Lambda =8\rho _{NP}$, embedded into an ITO host matrix on top of an a:Si substrate.

**Figure 6:**
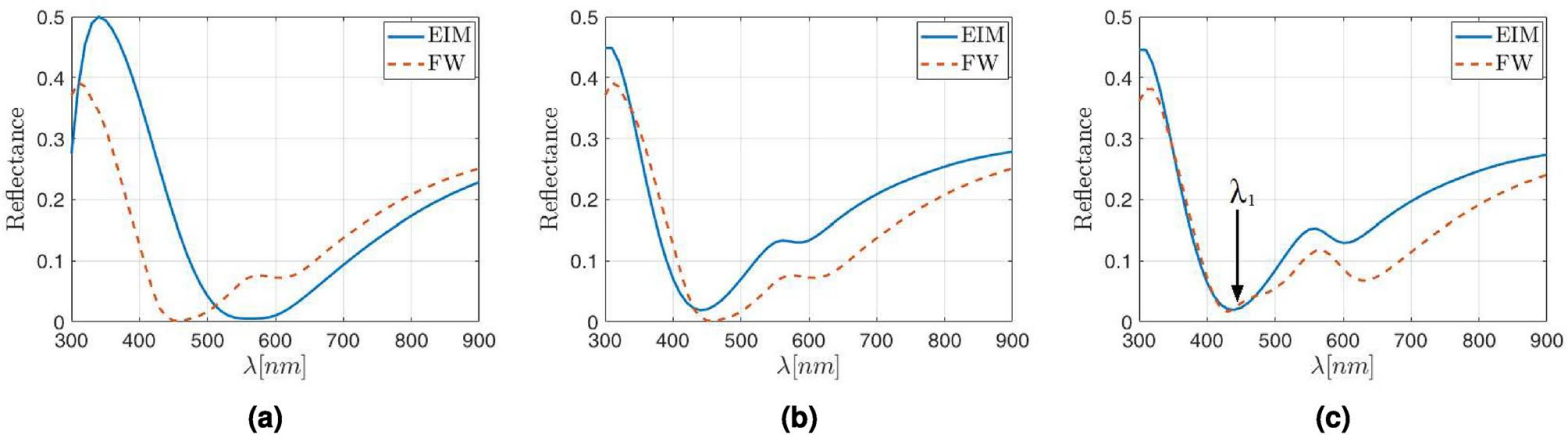
Reflectance obtained with FW (red dashed line) and neff (blue solid line) for Ag NP 30 nm radius inside an ITO host matrix with (**a**) lattice parameter of $\Lambda =8\rho _{\text{NP}}$ and $\Gamma =1$ (**b**) lattice parameter of $\Lambda =8\rho _{\text{NP}}$ and (**c**) $\Lambda =6\rho _{\text{NP}}$. In both cases (**b**) y (**c**) a thickness correction factor of $\Gamma =0.85$ has been applied when using the effective index model.

The reflectance calculated using the EIM is again spectrally shifted with respect to the results obtained with a FW package. This effect is shown in Fig. 6a, where the FW reflectance is plotted in red solid line while the obtained with the equivalent structure appears as a dashed blue line. Thus, a further correction of the effective thickness was made following the same criterion applied in “Dielectric NPs embedded into an ITO matrix”. In this case, although the radius of the nanoparticle changes $\Gamma$, its dependence with the radius is linear and lies in the interval [0.8, 0.9]. Therefore, we select a value $\Gamma =0.85$, which represents the average correction factor when considering the spectral shift and the area between the reflectance curves for both methods. After applying this new correction factor, the obtained reflectances are plotted in Fig. 6b, where the comparison between the results released by both methods is shown. Similar results are shown in Fig. 6c for a reduced lattice parameter equal to $\Lambda =6\rho _{\text{NP}}$.

As observed in Fig. 6, the shape of the reflectance is quite similar for both curves. However, they show larger differences with respect to the case of dielectric NPs. Even though, the average error remains below 10$\%$. Furthermore, a local increase in reflectance located around $\lambda = 570$ nm is qualitatively predicted by the EIM in the case of diluted media with surface coverage up to 9%. As can be also seen in this later case, a second local maximum is beginning to emerge when the lattice parameter is $\lambda =6\rho _{NP}$, and it is marked with an arrow in Fig. 6b.

**Figure 7:**
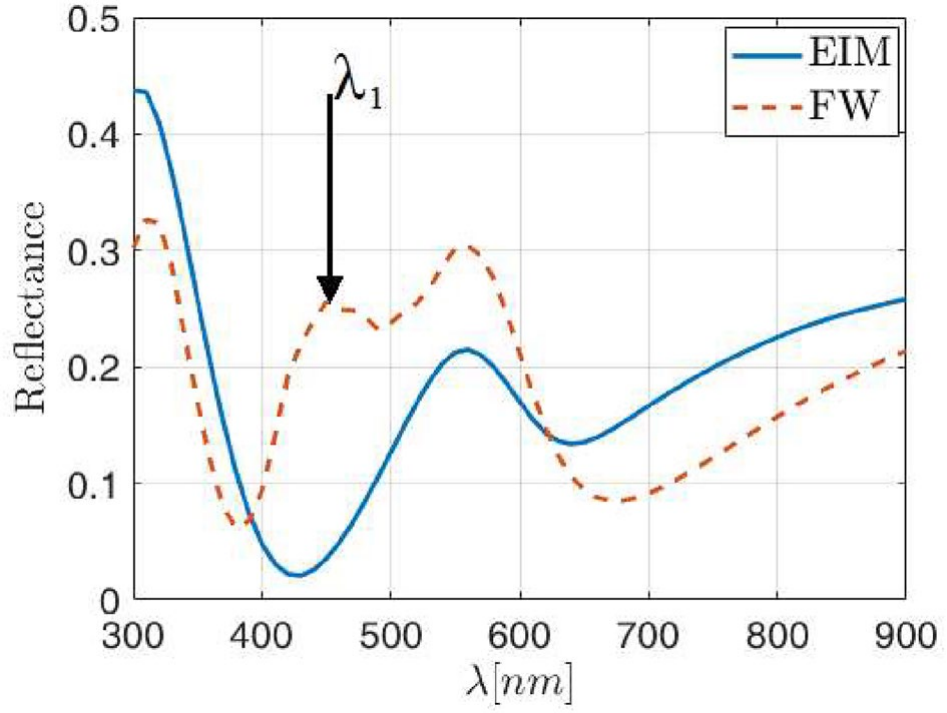
Reflectance obtained with FW (red dashed line) and the EIM (blue solid line) for Ag NP of 30 nm radius inside an ITO host matrix with lattice parameter of $\Lambda =4\rho$ and thickness correction factor of $\Gamma =0.85$.

However, a catastrophic failure of the effective index model appears when the lattice parameter decreases to $\lambda =4\rho _{\text{NP}}$, which would mean a surface coverage of about 20$\%$, far beyond of what can be considered dilute medium, even considering small NPs with $\rho _{\text{NP}}=30$ nm radius. These errors have been widely discussed in the literature, with numbers up to 50$\%$ at certain frequencies^52,53^, similar to those in Fig. 7. In general, it is observed that the DDA error strongly depends on the size and shape of the NPs, and in the case of metallic NPs—where the interactions between them are very important—the reliability of the results decreases when the distance between NPs decrease^54^. Indeed, the second local maximum marked in Fig. 6c, becomes relevant enough to be comparable with the main reflectance maximum, as shown in Fig. 7. From these results, the main features of the reflectance provided by the simple EIM qualitatively agree with those given by the FW method, assuming that the calculation is done within the limits of low wavelengths (below 380 nm) and large ones (beyond 500 nm). If this happens, the differences are still below a 10$\%$, but a new intense reflectance contribution at $\lambda _1$ in Fig. 7 appears in the intermediate range. This contribution cannot be corrected by simply applying the effective thickness correction, which in this figure is still a constant value of $\Gamma =0.85$.

**Figure 8:**
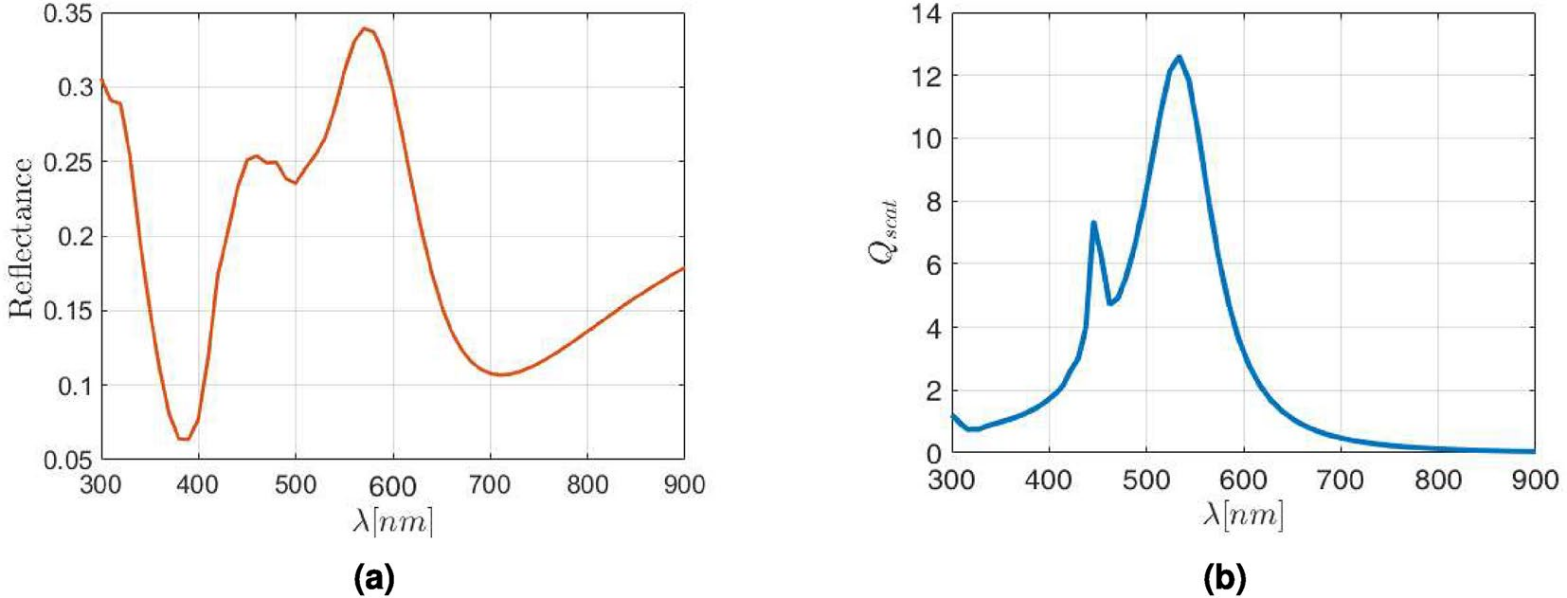
Reflectance obtained with FW for Ag NP 30 nm radius inside an ITO host matrix with lattice parameter of $\Lambda =4\rho _{\text{NP}}$ (**a**), and the scattering efficiency of an equivalent normal distribution (**b**) obtained from the Mie theory^55^.

The difference in results comes from the interaction between the electromagnetic fields in the NP surroundings and the excited LSPR. In this case a nanoantenna effect enhances the scattering and absorption properties of the NPs, being this effect greater as larger the nanoparticle is. This behavior is compatible with the scattering of silver NPs into an ITO matrix since the scattering is mainly forward at low wavelengths, which increases the absorption probability inside the metasurface itself, and therefore reduces the overall reflectance at these wavelengths. In fact, if the scattering efficiency of an equivalent distribution of 30 nm in radius silver NPs embedded in ITO is analyzed using the Mie theory^55^, a good qualitative agreement is observed for the two local maxima of the reflectance provided by the FW calculus, as can be seen in Fig. 8. But there is an energy transfer from the strongest scattering at $\lambda =560$ nm towards the secondary scattering maximum at $\lambda =450$ nm due to the lattice contribution to the global reflectance. In fact, the main scattering contribution is qualitatively predicted also by the simple EIM, losing the secondary one due to the lack of multipolar interactions considered in such model. This behavior is repeated for other NP radius and area’s coverage. On the other hand, at intermediate wavelengths, the backward scattering increases with respect to forward one, which explain the increase in reflectance.

**Figure 9:**
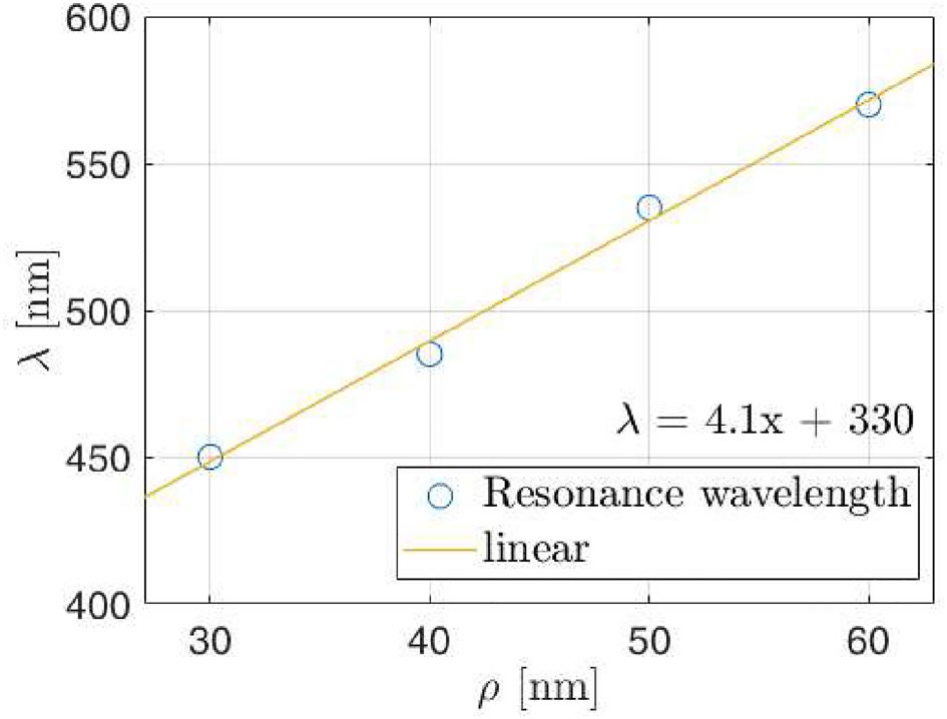
Values of the additional resonance wavelength $\lambda _1$(open circles) as a function of the NPs radii, for a square arrangement of Ag NPs embedded in ITO with lattice parameter $\Lambda =4\rho _{\text{NP}}$. The linear fit is also plotted in solid yellow line, showing a slope of $\Lambda$.

Up to this point, we can see major discrepancies between the FW and the effective medium model only when the lattice parameter falls below $\Lambda =6\rho _{\text{NP}}$ (around 9% surface coverage). This happens beyond the tolerance limit of the DDA approach, and affects the capability to faithfully obtain an effective index of the equivalent layer, once the effective thickness has also been corrected. Further inspection of this behavior is related with the evolution of the spectral reflectance with the NP radius. As a general rule, the spectral shift depends on the NP’s radius, $\rho _{\text{NP}}$, and also on the lattice parameter, $\Lambda$, as the interaction between NPs becomes more relevant. If we now look at the local maximum $\lambda _1$ (see Fig. 7)—where the main difference between both models appears, and analyze its evolution we can see that, keeping $\Lambda =4\rho _{NP}$, $\lambda _1$ moves to $\lambda _1=450$ nm for $\rho _{\text{NP}}=30$ nm or $\lambda _1=570$ nm for $\rho _{\text{NP}}=60$ nm radius Ag NPs, we find that the slope of such shift coincides with the lattice parameter, which can be written as

4 $$\begin{aligned} \lambda _1\left( \rho _{NP}\right) =\Lambda \rho _{NP}+330 \text{ nm } , \end{aligned}$$

and is plotted in Fig. 9. And furthermore, the spectral separation between the two local maxima is linear with the NP radius itself. Actually, this separation can be written as:

5 $$\begin{aligned} \Delta \lambda =\Lambda \rho _{NP}+90 \text{ nm }. \end{aligned}$$

This dependence is obtained even though the EIM model cannot predict that resonance by adjusting the effective thickness. This behavior is also fulfilled for lattice parameter $\Lambda =5\rho _{NP}$ and $\Lambda =3\rho _{NP}$, which reveals a lattice dependence of the spectral reflectance when the arrangement is compact enough—possibly related to the grating effect, although no mathematical derivation has been found that can represent this behavior exactly, even though it is an effect that has already been detected in other works^53,54^. From Fig. 6 we can prove that the reflectance from a metasurface with a lattice parameter of $\Lambda =6\rho _{NP}$ can also be adequately predicted through the EIM with an average accuracy below 10$\%$, supporting the applicability of it. Indeed, EIM is useful for metasurfaces based on spherical NPs embedded into a host matrix for almost any lattice parameter and NP radii when the NPs are dielectric, but only for surface coverage below 10$\%$ when the NPs are metallic, although even this ratio is higher than many of the designs presented in the literature^31,34^. In any case, the lattice dependence of the reflectance spectral shift for metallic NP paves the way to improve the model accuracy and applicability by adding new terms to the reflectance claculations, such as the inclusion of quadrupoles or terms to compensate for the grating effect.

## Conclusions

In this work, we have evaluated the reflectance under normal incidence of a metasurface made as a square array of spherical nanoparticles embedded into a host matrix. Our analysis uses an analytical model, the Effective Index Model, based on the replacement of the metasurface by an equivalent homogeneous layer. The results have been positively compared with those obtained from FW method based on FEM. When compared the reflectance calculated by both methods, the EIM agrees well with the FW results with an average difference below 5% when the NPs are dielectric. This agreement is maintained even if the surface coverage is around 20%, beyond the reported results up to date, with NPs radii up to 60 nm. Similar results can be obtained when the NPs are metallic ones. However, the surface coverage for an average error below 10$\%$ is limited to about 9$\%$. Beyond this value, the analytical model fails in reproducing some other important features in the spectral reflectance. The EIM approach predicts a maximum reflectance at a lower wavelengths larger than the one when obtained through the FW calculation. These discrepancies originates from the scattering properties of metallic NPs arrays, which strongly interact with LSPR.

Therefore, based on the obtained results, the applicability of the effective index model is numerically demonstrated for metasurfaces based on dielectric NPs with radii up to 60 nm and lattice parameter of $\Lambda =4\rho _{\text{NP}}$, which represents a surface coverage of about 20%, beyond the value reported up to date for square arrays. Our calculation limits the observed error below 5$\%$ when compared with the very same arrangements studied through FW simulations, once the effective layer thickness is also corrected. When the NPs are metallic, the simple EIM is limited to the case when the lattice parameter is greater or equal to $\Lambda =6\rho _{\text{NP}}$, which means a surface coverage of about 9%, still beyond the usual value reported in literature. The observed dependence of the reflectance with the lattice parameter, can be used to include additional terms in the effective index model to improve its performance. Finally, it should be noted that one of the biggest advantages of the EIM compared to traditional FW is the computing time. As it is a simple model, its performance is especially good, obtaining a minimum of 99$\%$ reduction in execution time compared to FW evaluation. For more information, consult Supplementary Tables S1 and S2.

## Supplementary Information

Supplementary Information.

## Data Availability

All data and code used to generate the results presented are available from the corresponding authors upon request.
