# Supplementary material for: Effective index model as a reliable tool for the design of nanostructured thin-film solar cells
Source: Sci Rep. 2023 Apr 17;13:6227. doi: 10.1038/s41598-023-33085-3 (PMC10110609; doi:10.1038/s41598-023-33085-3)
Supplement: Supplementary file 1 — Supplementary Information. [file 41598_2023_33085_MOESM1_ESM.pdf]

The Discrete Dipole Approximation

The effective optical properties to be used in reflectance calculations depend on the dielectric permittivity components of the metasurface that can be written as[44]:

$$\varepsilon^{\parallel}(\lambda) - \varepsilon_{\text{host}}(\lambda) = \frac{4\pi}{\Lambda^2 d}\alpha^{\parallel}(\lambda), \tag{S1}$$

$$\frac{1}{\varepsilon^{\perp}(\lambda)} - \frac{1}{\varepsilon_{\text{host}}(\lambda)} = \frac{4\pi}{\Lambda^2 d}\frac{\alpha^{\perp}(\lambda)}{\varepsilon^2_{\text{host}}(\lambda)}, \tag{S2}$$

where Λ is the 2D lattice constant, $\varepsilon_{\text{host}}$ is the wavelength dependent dielectric function of the environment where the NPs are embedded, d is the effective thickness of the metamaterial layer and $\alpha^{\perp}, \alpha^{\parallel}$ are the effective polarizability, either perpendicular or parallel to the interface, of the NP interacting with all the other NPs in the array. These polarizabilities are expressed as a function of the individual NP one[44]:

$$\alpha^{\parallel}(\lambda) = \frac{\alpha(\lambda)}{1 + \frac{\alpha(\lambda)}{\varepsilon_{\text{host}}(\lambda)}\left[-\frac{1}{2}\frac{U_A}{\Lambda^3} + \xi(\lambda)\left(\frac{f(h,\Lambda)}{\Lambda^3} - \frac{3}{2}\frac{g_1(h,\Lambda)}{\Lambda^3} + \frac{1}{8h^3}\right)\right]}, \tag{S3}$$

$$\alpha^{\perp}(\lambda) = \frac{\alpha(\lambda)}{1 + \frac{\alpha(\lambda)}{\varepsilon_{\text{host}}(\lambda)}\left[\frac{U_A}{\Lambda^3} - \xi(\lambda)\left(\frac{f(h,\Lambda)}{\Lambda^3} - 12\frac{g_2(h,\Lambda)}{\Lambda^5} + \frac{1}{4h^3}\right)\right]}. \tag{S4}$$

Here, $\alpha^{\parallel}, \alpha^{\perp}(\lambda)$ are the polarizabilities of the single NP, $\xi(\lambda)$ is the wavelength-dependent image-charge screening factor, U_A is a lattice-dependent parameter, and $f(h,\Lambda), g_1(h,\Lambda)$ and $g_2(h,\Lambda)$ are lattice-dependent functions that include the moduli of all the possible vectors in the 2D plane array casculated for a square lattice as:

$$U_A = \sum_i \sum_j \frac{1}{(i^2 + j^2)^{3/2}}, \tag{S5}$$

$$f(h,\Lambda) = \sum_i \sum_j \frac{1}{(i^2 + j^2 + (\frac{2h}{\Lambda})^2)^{3/2}}, \tag{S6}$$

$$g_1(h,\Lambda) = \sum_i \sum_j \frac{(i^2 + j^2)}{(i^2 + j^2 + (\frac{2h}{\Lambda})^2)^{5/2}}, \tag{S7}$$

$$g_2(h,\Lambda) = \sum_i \sum_j \frac{1}{(i^2 + j^2 + (\frac{2h}{\Lambda})^2)^{5/2}}, \tag{S8}$$

where h is the distance from the NP center to the active layer, $h = d_{\text{phys}}/2$, with the sums converging for a $j \approx 2 \times 10^4$, which is the estimated value for a low error in scattering calculations[53]. Thus, for a square lattice, $U_A = 9.0331, U_B = 5.0903$ and $U_C = 4.4231$; while for a hexagonal lattice $U_A = 11.0334, U_B = 6.7618$ and $U_C = 6.1951$. As said above, a further correction must be made to Eqs. (S3) and (S4). When the NP size is beyond the Rayleigh limit (radius larger of about 50 nm)[38], the quasi-static approximation is no longer valid[39,40,54]. Instead, this polarizability needs to be modified to include dynamic depolarization and radiative damping effects. This correction is known as the Modified Long-Wavelength Approximation (MLWA), and can be written as:

$$\alpha_{\text{MLWA}}(\lambda) = \frac{\alpha_{\text{static}}(\lambda)}{1 - \frac{2}{3}ik^3\alpha_{\text{static}}(\lambda) - \frac{k^2}{l_E}D\alpha_{\text{static}}(\lambda)}, \tag{S9}$$

where k is the wave vector of the medium and $\alpha_{\text{static}}(\lambda)$ is the electrostatic polarizability, described as [39] :

$$\alpha_{\text{static}}(\lambda) = \frac{V}{4\pi} \frac{\varepsilon_{\text{NP}}(\lambda) - \varepsilon_{\text{host}}(\lambda)}{3\varepsilon_{\text{host}}(\lambda) + 3L(\varepsilon_{\text{NP}}(\lambda) - \varepsilon_{\text{host}}(\lambda))}. \tag{S10}$$

The parameters of the NP appear in Eqs. (S9) and (S10). Thus, D is the dynamic geometrical factor[38], l_E is the NP semi-axis, V is the NP volume and L is the NP shape factor[38], which depends on the ratio between major and minor axis of an ellipsoid NP with revolution symmetry. In our case, we restrict our study to spherical NPs, therefore, $D = 1, l_E = \rho_{\text{NP}}$, and $L = 1/3$. Otherwise, the correct values can be calculated from[38,55] as a function of the orientation of the incident electric field. Finally, the absorption, extinction and scattering cross sections, σ_{abs}, σ_{ext} and σ_{scat}, are defined as[39]:

$$\sigma_{\text{abs}} = 4\pi k Im(\alpha_{\text{MLWA}}), \tag{S11}$$

$$\sigma_{\text{scat}} = \frac{8}{3}\pi k^4 |\alpha_{\text{MLWA}}|^2, \tag{S12}$$

$$\sigma_{\text{ext}} = \sigma_{\text{abs}} + \sigma_{\text{scat}}, \tag{S13}$$

Computational Time

The physical mechanism involved in the FW approach is the numerical evaluation of the Maxwell's equations within each finite element meshed in the computational cell. The physical mechanism behind the EIM is the application of the boundary conditions at the interfaces of the structures that are modeled as having geometrical (height) and material (effective index) parameters obtained from an evaluation of the mix between the matrix element and the nanoparticles. All Computationals Time (CT) have been obtained by performing simulations for the reflectance. Table S1 shows the times for tests varying the rho. The EIM model maintains constant times, while the FW times are directly proportional to the size of the NP. On the other hand, Table S2 shows the times obtained for a rho scan of $[30 - 60]nm$, being in these simulations with iterations where the EIM model is especially useful. All the tests are carried out in the same conditions on a computing server with a CPU Intel Xeon 3.50 GHz, 128 GB RAM and an 8 GB NVIDIA Quadro RTX 4000, running under Windows 10.

	CT [s]			
Model	ρ_{NP} 30 nm	ρ_{NP} 40 nm	ρ_{NP} 50 nm	ρ_{NP} 60 nm
EIM	1.9	2	2.1	2
FW	474	879	1254	2980

Table S1. CT comparison between the EIM model and the FW for different NP radii.

Model	CT [s]
EIM	174
FW	11783-450000

Table S2. CT comparison between the EIM model and the FW for one-parameter sweeps.

Other materials

Comparing Figures S1 and S2, we obtain for AlN and Au the same conclusions already presented in the paper for SiO_2 and Ag. The dielectric materials present an almost exact similarity between both models, even in the most compact values (such as that of Figure S1, with a lattice value of $\Lambda = 4\rho_{\text{NP}}$). The metallic materials present the greatest difference, which also increases when the structure becomes more compact (due to the interaction between NP and the appearance of quadrupole effects) as observed in the comparison of Figures S2a and S2b. In fact, Figure S2b shows the appearance, at 550nm, of the same secondary resonance peak as in the case of silver. Peak that, as in the example explained in the article, the EIM model is unable to calculate.

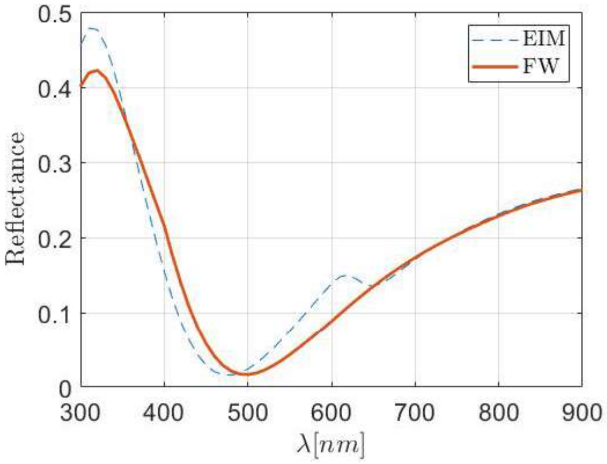

Figure S1. Reflectance obtained with FW (red dashed line) and EIM (blue solid line) for an 30nm AlN NP, with a lattice parameter of $\Lambda = 4\rho_{\mathrm{NP}}$ and a thickness correction factor $\Gamma = 0.85$

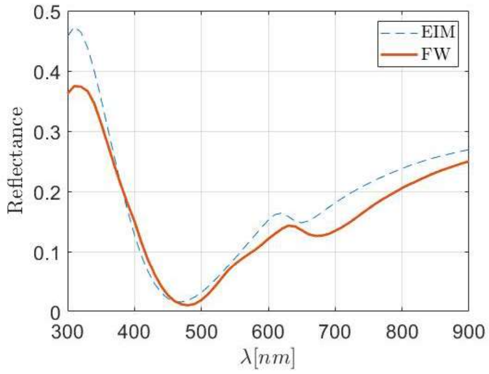

(a)

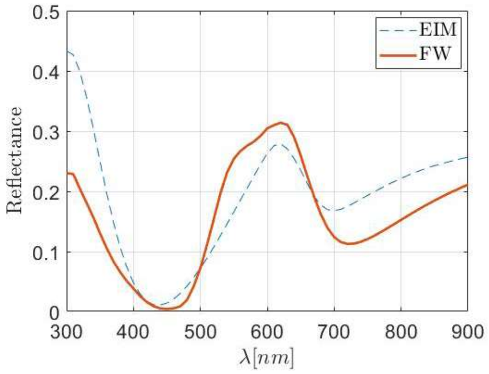

(b)

Figure S2. Reflectance obtained with the FW (red dashed line) and EIM (blue solid line) for an 30nm Au NP when (a) the lattice parameter is $\Lambda = 8\rho_{\mathrm{NP}}$. (a) the lattice parameter is $\Lambda = 4\rho_{\mathrm{NP}}$. Both cases with the correction factor $\Gamma = 0.87$ applied to the physical thickness of the metasurface layer.
